# Imperforate Hymen: A Report of a Case With Classical Signs

**DOI:** 10.7759/cureus.56014

**Published:** 2024-03-12

**Authors:** Rasika D Zade, Amruta Choudhary, Saunitra A Inamdar, Nandkishor J Bankar, Mugdha Junghari

**Affiliations:** 1 Obstetrics and Gynaecology, Datta Meghe Medical College, Datta Meghe Institute of Higher Education and Research, Wardha, IND; 2 Obstetrics and Gynaecology, All India Institute of Medical Sciences, Nagpur, IND; 3 Microbiology, Jawarhal Nehru Medical College, Datta Meghe Institute of Higher Education and Research, Wardha, IND

**Keywords:** hymenotomy, hymenectomy, female external genitalia, cyclical pelvic discomfort, hematometra, hematocolpos, imperforate, hymen

## Abstract

An imperforate hymen (IH) is a condition where the hymen, which is a thin membrane that partially covers the vaginal opening, completely obstructs the vaginal canal. This condition is associated with problems such as pelvic mass, cyclical abdominal discomfort, and difficulty in urination. The occurrence of IH is quite rare, with an incidence of only one in 1000-10,000 women worldwide. We discuss a classical case of primary amenorrhoea with associated complaints of urinary retention and its management by hymenotomy (cruciate incision). We also considered the risk of hymen re-closure due to the lack of estrogenization of genital tissue and offered the patient the option of vaginal molding.

## Introduction

The vaginal introitus is covered by a delicate layer of stratified squamous epithelium known as the hymen. Imperforate hymen (IH) is a condition where this thin membrane, partially covering the vaginal opening, completely obstructs the vaginal canal [1]. IH is an uncommon hindrance affecting the female reproductive system and is associated with issues such as pelvic mass, cyclical abdominal discomfort, and difficulty in urination [2,3]. The occurrence of IH is rare, with only one in 1000-10,000 women affected globally [3]. It is crucial to conduct a thorough examination of the external genitalia of teenage females experiencing urinary retention, primary amenorrhea, or cyclical pelvic discomfort to eliminate the possibility of this ailment, ensuring no details are overlooked [4].

Teenagers often do not exhibit symptoms until menarche, when they may experience amenorrhea with cyclic abdominal pain. If a pelvic mass appears as a bulging perineal mass that is blue in color during a physical examination, it is referred to as hematometra or hematocolpos [5-7]. The accumulation of menstrual blood in the vaginal or uterine areas can lead to these conditions, causing a physical blockage in the urinary system and issues such as acute renal injury, hydronephrosis, or urinary retention [8]. If a teenage girl is not facing any severe pelvic mass problems requiring immediate surgery to preserve kidney function, address an infection, or reduce infertility, it is advisable to postpone corrective hymenectomy until adolescence. Surgical treatment is the method of choice to avoid the possibility of IH re-closure [9,10].

## Case presentation

**Figure 1:**
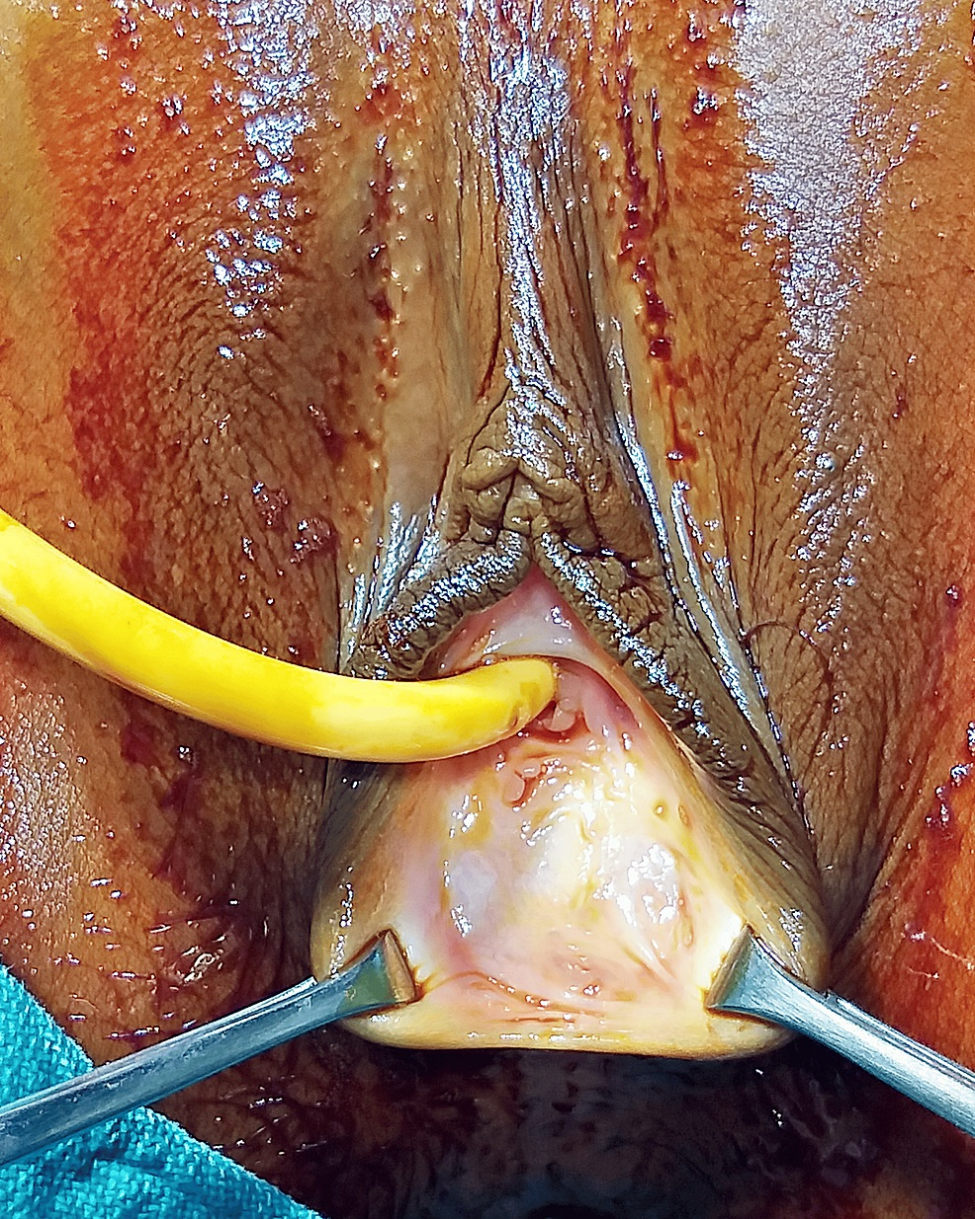
Imperforate hymen

A 15-year-old adolescent girl presented to the Obstetrics and Gynecology (OBGY) outpatient department (OPD) accompanied by her mother, complaining of primary amenorrhea and cyclical abdominal pain for the past two years. Additionally, she reported difficulty in urination over the last five days, leading to acute urinary retention. Two days prior, she had been catheterized at the Government Hospital due to the same issue. Acute urinary retention is commonly associated with hematocolpometra. To investigate her complaints, a micturating cystourethrogram (MCU) was performed to rule out any urinary tract anomalies. The MCU study revealed no radiographic structural abnormalities, no evidence of reflux, and a normal urethra without apparent intrinsic or extrinsic filling defects. Subsequently, a pelvic ultrasound was performed, revealing a markedly dilated vagina containing thick echogenic fluid (likely old blood), indicative of hematocolpos secondary to IH. During the physical examination, a perineal tense bulge with a bluish hue was observed at the introitus (Figure 1). The patient was catheterized once again to alleviate acute urinary retention, which resulted from the obstruction of the urinary tract by hematocolpos.

**Figure 2:**
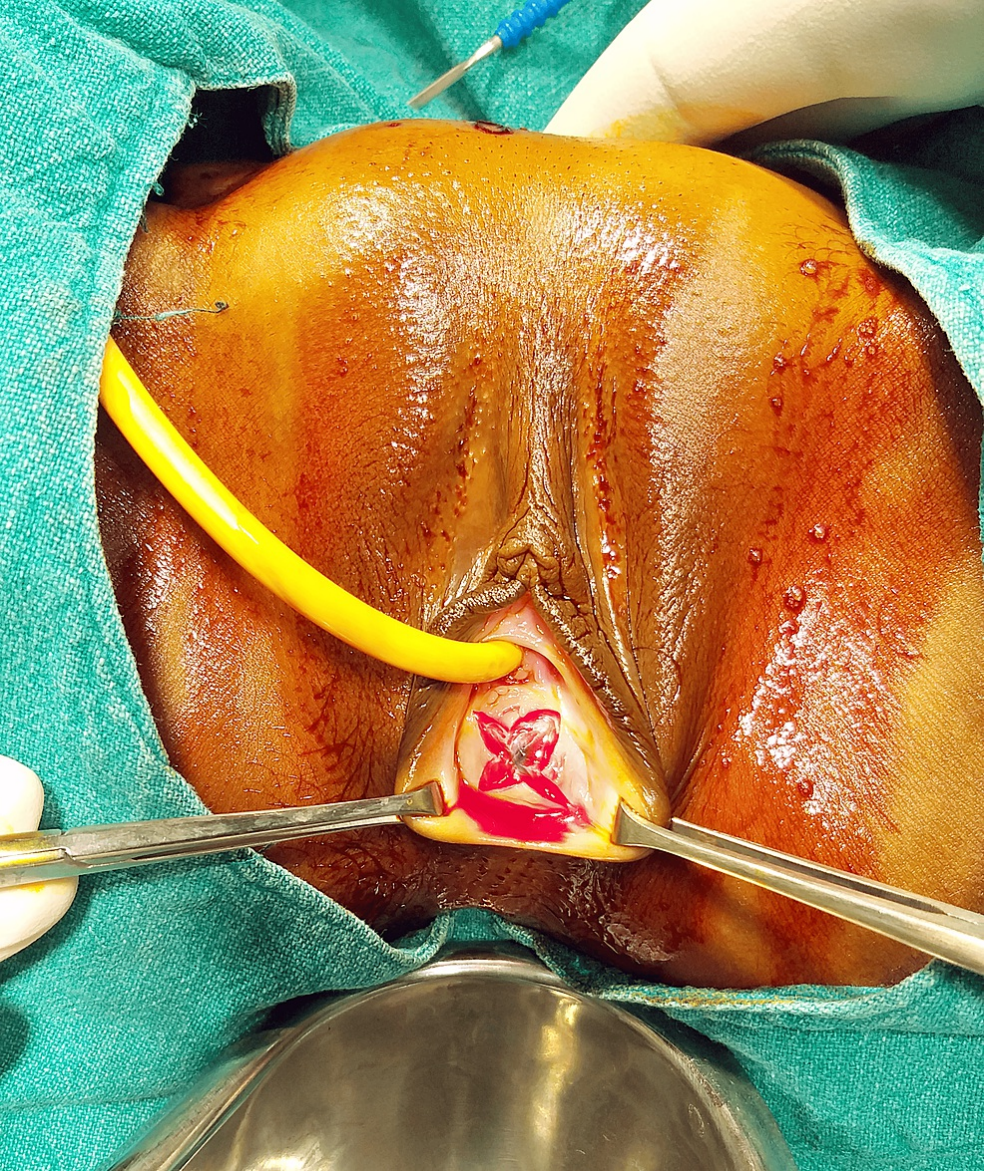
Cruciate incision over imperforate hymen

**Figure 3:**
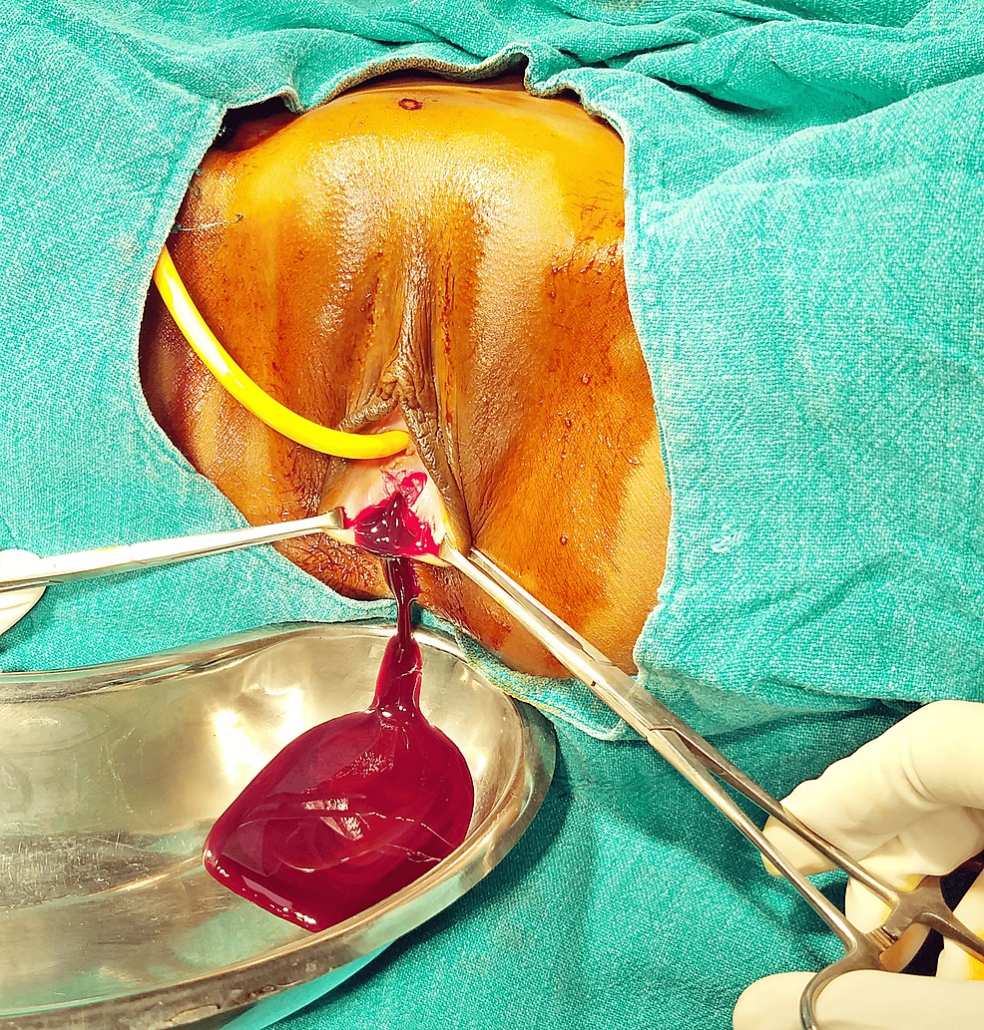
Drainage of hematocolpos

We confirmed her diagnosis by systematically eliminating other potential causes. During her pre-anesthetic check-up, her blood analysis revealed severe anemia with a hemoglobin level of 5.9 g%. Consequently, we promptly scheduled her for the procedure and conducted a hymenectomy, employing a cruciate incision on the hymen (Figure 2), followed by drainage of the sizable hematocolpos (Figure 3). Subsequently, one unit of packed red cells (PRC) was transfused. An MRI of the pelvis was also recommended to rule out any other Müllerian anomalies, but it yielded normal results. A follow-up visit was scheduled after one month, during which she reported normal and regular menstruation. Additionally, counseling was provided concerning vaginal molding.

## Discussion

In a study involving 236 postnatal patients with an average age of 10.7 ± 4.7 years, the patients presented with various symptoms, including urinary retention, abdominal pain, dysuria, abnormal menstruation, increased urinary frequency, severe renal failure, and urinary tract infection. Surgical treatment was the most common option for patients diagnosed with the condition, with many undergoing hymenotomy and hymenectomy. Only seven patients received prophylactic antibiotics as they were at risk of ascending pelvic infections [8]. Both surgical methods produced similar outcomes, and IH may be linked to additional developmental Müllerian anomalies [11]. Although some reports have suggested that Müllerian anomalies may not always be linked to it, it is still unclear whether evaluating urogenital anomalies is necessary. However, it is believed that a thorough evaluation of all possible anomalies is always beneficial for ensuring optimal health outcomes [12].

The iatrogenic pelvic inflammatory disease has been reported in a patient following the hymenotomy procedure, which was carried out under sterile conditions. The surgical area had sealed by itself within a day, and a gynecologist examined the female, who was later scheduled for a hymenectomy in the operating room two days later. On the seventh day post-surgery, the patient experienced a high-grade fever and pain in the lower abdomen. Further examination via MRI revealed that the patient had developed right pyosalpinx and required CT-guided drainage. Therefore, it is important to avoid even minor incisions in an IH for diagnosis or relief of symptoms due to the risk of iatrogenic infection caused by bacterial contamination in the hematocolpos [13].

If a patient requires immediate hymenectomy because of an infection or urinary tract blockage, it is crucial to perform the procedure in an operating room in a sterile environment. The risk of hymen re-closure is believed to be higher in prepubertal patients because their genital tissue lacks estrogen [14]. If the hymenectomy is not done adequately, it can lead to stenosis and closure, which can cause dyspareunia and infertility [15].

## Conclusions

It is crucial to thoroughly investigate any adolescent female who complains of abdominal pain and urinary retention, especially if she has not yet started menstruating. The presence of hematocolpos or IH must be ruled out with the assistance of a team of medical professionals, including pediatricians, gynecologists, and pediatric urologists. Identifying and treating an IH early can prevent serious short-term and long-term complications such as acute kidney injury and peritonitis. A female suspected of having an IH must undergo a primary investigation involving abdominal and pelvic ultrasound scans, which should also include an assessment of the volume of hematocolpos or hematometra. Although not studied to date, there appears to be a correlation between the volume of hematocolpos or hematometra and the presenting signs and symptoms. This correlation is likely directly proportional, indicating that the severity of signs in a patient can be inferred from the volume of hematocolpos or hematometra.
